# Prey availability and intraguild competition regulate the spatiotemporal dynamics of a modified large carnivore guild

**DOI:** 10.1002/ece3.7620

**Published:** 2021-05-16

**Authors:** Robert S. Davis, Richard W. Yarnell, Louise K. Gentle, Antonio Uzal, William O. Mgoola, Emma L. Stone

**Affiliations:** ^1^ School of Animal, Rural and Environmental Sciences Nottingham Trent University Brackenhurst Campus Southwell UK; ^2^ Conservation Research Africa & Carnivore Research Malawi Lilongwe Malawi; ^3^ Department of National Parks and Wildlife Malawi Lilongwe Malawi; ^4^ Department of Applied Sciences University of the West of England Bristol UK

**Keywords:** activity patterns, camera traps, carnivore ecology, competition, niche segregation, predator dynamics

## Abstract

Effective conservation management requires an understanding of the spatiotemporal dynamics driving large carnivore density and resource partitioning. In African ecosystems, reduced prey populations and the loss of competing guild members, most notably lion (*Panthera leo*), are expected to increase the levels of competition between remaining carnivores. Consequently, intraguild relationships can be altered, potentially increasing the risk of further population decline. Kasungu National Park (KNP), Malawi, is an example of a conservation area that has experienced large‐scale reductions in both carnivore and prey populations, leaving a resident large carnivore guild consisting of only leopard (*Panthera pardus*) and spotted hyena (*Crocuta crocuta*). Here, we quantify the spatiotemporal dynamics of these two species and their degree of association, using a combination of co‐detection modeling, time‐to‐event analyses, and temporal activity patterns from camera trap data. The detection of leopard and spotted hyena was significantly associated with the detection of preferred prey and competing carnivores, increasing the likelihood of species interaction. Temporal analyses revealed sex‐specific differences in temporal activity, with female leopard activity patterns significantly different to those of spotted hyena and male conspecifics. Heightened risk of interaction with interspecific competitors and male conspecifics may have resulted in female leopards adopting temporal avoidance strategies to facilitate coexistence. Female leopard behavioral adaptations increased overall activity levels and diurnal activity rates, with potential consequences for overall fitness and exposure to sources of mortality. As both species are currently found at low densities in KNP, increased risk of competitive interactions, which infer a reduction in fitness, could have significant implications for large carnivore demographics. The protection of remaining prey populations is necessary to mitigate interspecific competition and avoid further alterations to the large carnivore guild.

## Graphical abstract

Carnivore guild dynamics can be altered by changes in community assemblage and the loss of natural prey. Here, we quantify the spatiotemporal dynamics of leopard and spotted hyena in a modified carnivore guild where they are the only remaining resident members. The presence of leopard and spotted hyena was significantly associated with the detection of preferred prey and competing carnivores, increasing the likelihood of species interaction. Female leopard exhibited temporal responses to mitigate potential conflict with spotted hyena and male conspecifics.

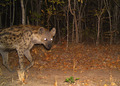

## 1 INTRODUCTION

Global environmental change is driving the decline in large carnivore populations and can be attributed to numerous factors, including habitat destruction, loss of natural prey, reduced landscape connectivity, and human–wildlife conflict (Ripple et al., 2014; Wolf & Ripple, 2016). Rising anthropogenic impacts increase pressure on species interactions through the loss of complex carnivore guilds, declines in natural prey, and shrinking protected area networks (Jones et al., 2018; Sévêque et al., 2020). These factors can distort carnivore dynamics and ecosystem function through increased competition for resources (Creel et al., 2018; Manlick & Pauli, 2020), reduced suppression of mesocarnivores (Brook et al., 2012; Prugh & Sivy, 2020), shifts in spatial use (Carter et al., 2019; Parsons et al., 2019), and changes in survival rates for dominant and subordinate competitors (M’soka et al., 2016; Elbroch & Kusler, 2018). These alterations in community assemblage and species dynamics can result in cascading trophic effects (Finke & Denno, 2005; Suraci et al., 2016; Winnie & Creel, 2017). As large carnivore dynamics have a key regulating effect on density and resource partitioning (Dröge et al., 2017; Groom et al., 2017), understanding their ecological and anthropogenic drivers is critical for effective conservation management (Davis et al., 2018; Sévêque et al., 2020).

The spatiotemporal dynamics of large carnivores have been widely investigated across sub‐Saharan Africa (e.g., Balme et al., 2019; Dröge et al., 2017; Hayward & Slotow, 2009; Rafiq, Jordan, Wilson, et al., 2020). However, few studies have examined the spatiotemporal dynamics of these species in habitats where competing guild members, most notably lion (*Panthera leo*), have been extirpated (M’soka et al., 2016). Lions are often the dominant competitor in African carnivore guilds, but due to their preference for larger prey items (>200 kg; Hayward & Kerley, 2005), tendency for livestock predation, and social nature, they are often at greater risk of localized extinction than other large carnivores (Everatt et al., 2019), such as leopard (*Panthera pardus*) and spotted hyena (*Crocuta crocuta*, hereafter hyena). In the absence of lions, interference competition between remaining members of the carnivore guild is predicted to intensify, which could lead to changes in dynamics and increase the risk of population decline (Périquet et al., 2015; M’soka et al., 2016). Large carnivore behavior is further driven by “bottom‐up” processes, of which the abundance and distribution of preferred prey are primary regulators (Hayward et al., 2007; Wolf & Ripple, 2016). As large carnivores often share a degree of dietary overlap, any decline in prey abundance is also likely to disturb species dynamics through increased competition for food or the concentration of carnivore activity in areas of higher prey availability (Creel et al., 2018).

How, and if, these altered environments impact species’ mechanisms of spatial use and temporal activity warrants further investigation. Malawi, in south‐central Africa, offers a unique opportunity to study carnivore dynamics. Widespread persecution and the depletion of large prey species have led to the localized loss of resident lion populations, with the species restricted to either infrequent dispersing males or small isolated populations in fenced reserves (Briers‐Louw et al., 2019; Davis et al., 2021; Mésochina et al., 2010). Malawi has one of the highest population densities in Africa (186 people/km^2^; National Statistical Office, 2019), with 80% of the population dependent on natural resources (e.g., firewood) and agriculture for income, heating, and food security (Yaron et al., 2011; Schaafsma et al., 2018). Subsequently, Malawi has the highest deforestation rate in Africa (Mapulanga & Naito, 2019), while protected areas have been subject to widespread subsistence poaching (van Velden et al., 2020). Kasungu National Park (KNP) is a model example of a protected area in Malawi that has experienced these declines in carnivore and prey populations (Davis et al., 2021; Munthali & Mkanda, 2002). As the second‐largest protected area in Malawi, comprised of miombo woodland, the primary habitat type across the country (Gondwe et al., 2019), and subject to the same environmental pressures as other reserves, KNP is a novel site to (a) test theories on resource and guild‐based competition, and (b) understand how species respond to anthropogenic disturbance.

The loss of a resident lion population means that leopard and hyena are the two dominant competitors in KNP. Both leopard and hyena are known to display wide habitat preferences, have diverse diets, and persist in areas of high human disturbance (Holekamp & Dloniak, 2010; Jacobson et al., 2016). These behavioral traits allow leopard and hyena to survive in areas where other apex predators cannot (Green et al., 2018; Loveridge et al., 2020). Localized extirpation of lion populations is expected to increase over the coming decades, with the species predicted to survive in only the largest protected areas across Africa and in small, intensively managed, reserves (Bauer et al., 2015). Consequently, understanding carnivore dynamics in areas of anthropogenic disturbance is important for predicting future alterations in carnivore guilds (Rafiq, Jordan, Wilson, et al., 2020). The intraguild dynamics of leopard and hyena in KNP can, therefore, act as a model to inform conservation management under increasing levels of environmental change.

Spatiotemporal dynamics between leopard and hyena are complex, with findings varying between habitats and carnivore community assemblages. The availability of preferred prey is known to significantly influence the presence of both species (Balme et al., 2019; Périquet et al., 2015; Searle et al., 2020). In addition, leopard kills are subject to high levels of kleptoparasitism from hyena (Balme et al., 2017), which is known to affect reproductive success in female leopards (Balme et al., 2013). Hyena are also a direct source of leopard mortality (Swanepoel et al., 2015). In some ecosystems, kleptoparasitism has resulted in leopard adopting either spatial (Comley et al., 2020; Ramesh et al., 2017) or temporal (Havmøller et al., 2020) avoidance strategies, although Ramesh et al. (2017) suggested that the spatial avoidance between leopard and hyena was due to lion presence. Leopards also exhibit behavioral adaptations (i.e., tree caching and dietary plasticity) to facilitate coexistence with hyena (Balme et al., 2019; Briers‐Louw & Leslie, 2020). However, the spatiotemporal dynamics of leopard and hyena are often overlooked (Rafiq, Jordan, Wilson, et al., 2020; Vanak et al., 2013), particularly in ecosystems where the carnivore guild has been depleted due to anthropogenic disturbance. The lack of understanding of coexistence strategies between leopard and hyena in such areas limits conservation management.

We used data from camera trapping surveys to investigate the spatiotemporal dynamics of leopard and hyena in KNP, a protected miombo woodland habitat where these species are the only remaining members of the large carnivore guild. We applied co‐detection modeling (Balme et al., 2019; Cusack et al., 2017), time‐to‐event analyses (Cusack et al., 2017), and temporal overlap comparisons (Rowcliffe et al., 2014) to evaluate the impact of a range of interspecific, ecological, and anthropogenic parameters on carnivore activity. The availability of preferred prey has previously been highlighted as a significant driver of leopard and hyena presence (Périquet et al., 2015; Searle et al., 2020), and accordingly, we predict that (a) the detection of both species will increase in relation to prey detectability, (b) this will result in significant rates of co‐detection between leopard and hyena, and (c) the potential for high levels of spatial overlap between leopard and hyena will result in leopard adopting temporal avoidance mechanisms to facilitate coexistence and avoid competition.

## 2 MATERIALS AND METHODS

### 2.1 Study site

**FIGURE 1:**
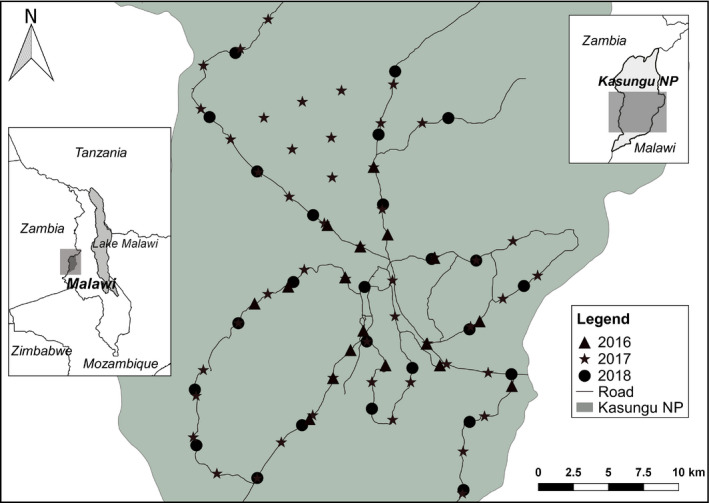
Camera trap locations for surveys conducted in 2016, 2017, and 2018 in Kasungu National Park, Malawi. Inset maps show the area covered within Kasungu National Park and the location of Malawi within sub‐Saharan Africa

Kasungu National Park (central coordinates S12.9092°, E33.1689°; Figure 1) is a 2,316‐km^2^ protected area in the central region of Malawi. KNP is dominated by miombo woodland, consisting of *Brachystegia* and *Julbernardia* spp. (Bhima et al., 2003). Closed canopy miombo woodland is interspersed with seasonally wet grassland areas (locally known as dambos) and isolated rocky inselbergs. The altitude ranges between 1,000 and 1,500 m, and mean annual rainfall is 780 mm (Bhima et al., 2003).

In the early 2000s, poaching was so prolific that populations of several remaining prey species were moved from KNP to Liwonde National Park, Malawi, as their survival could no longer be guaranteed in KNP (Munthali & Mkanda, 2002). Consequently, KNP has experienced a significant decline in large mammal (Bhima et al., 2003; Munthali & Mkanda, 2002) and carnivore populations (Davis et al., 2021). Lions, once known residents in KNP, are now restricted to dispersing individuals from the wider Malawi–Zambia Transfrontier Conservation Area (Davis et al., 2021; Mésochina et al., 2010), and cheetahs (*Acinonyx jubatus*), also previously known residents, have been extirpated (IUCN/SSC, 2015). While lions are not strictly extirpated from KNP, they are not present at levels that would have an influence on the guild dynamics of resident carnivore populations. Leopard and hyena are the only remaining resident large carnivore species in KNP, with densities in 2018 estimated at 1.77 leopard/100 km^2^ and 1.62 hyena/100 km^2^ (Davis et al., 2021).

### 2.2 Camera trap surveys

Data were collected from camera trap surveys lasting 90–120 days between May and October over a three‐year period (2016–2018; Figure 1). To maximize the detection probability of large carnivores, roads and major trails were prioritized for camera placement (Cusack et al., 2015; Davis et al., 2021). One camera was deployed per station, and stations were checked regularly to maintain camera function and data collection. All images were catalogued to species level, and individual leopards were sexed using criteria outlined in Henschel and Ray (2003).

### 2.3 Co‐detection modeling

We used a co‐detection modeling approach to assess predictors of leopard and hyena detection (Balme et al., 2019; Cusack et al., 2017). Due to high rates of naïve occupancy for both species, data were unsuitable for co‐occupancy analysis. The co‐detection approach allowed the use of data from all survey years. We measured the detection and nondetection of leopard and hyena as a binary response variable (“1” for detection, “0” for nondetection) for each camera trap station, using an occasion length of five days per sampling event. We chose the five‐day sampling event to correspond with the time frame for the time‐to‐event analysis (described below) and the low detection rates of both focal species resulting in zero inflation with a one‐day sampling occasion. Binary responses were modeled as a function of different combinations of detection covariates using binomial generalized linear mixed‐effect models (GLMMs; Bolker et al., 2009; Cusack et al., 2017).

Based on evidence from previous studies, we selected five covariates that could impact the likelihood of detection for both leopard and hyena, incorporating interspecific, environmental, and anthropogenic factors (Table 1). We measured prey detection from camera trap data as a binary response variable and assumed that prey species selected differed for leopard and hyena. As leopard diet in KNP has not been assessed, we selected known leopard prey species from a similar habitat type (Havmøller, Jacobsen, Havmøller, et al., 2020), or species for which we had anecdotal evidence (from camera traps and opportunistic kill sites) of predation in KNP. The following were included as leopard prey species: common duiker (*Sylvicapra grimmia*), bushbuck (*Tragelaphus sylvaticus*), bushpig (*Potamochoerus larvatus*), warthog (*Phacochoerus africanus*), yellow baboon (*Papio cynocephalus*), porcupine (*Hystrix africaeaustralis*), and savanna hare (*Lepus victoriae*). Preliminary diet analysis for spotted hyena in KNP identified common duiker, bushpig, savanna hare, warthog, bushbuck, and kudu (*Tragelaphus strepsiceros*) as the most frequent prey species, and as such, these species were selected for the hyena prey covariate (Carnivore Research Malawi, unpublished data).

**TABLE 1:** Detection covariates, with sampling range and mean, hypothesized to affect the likelihood of detection for leopard and spotted hyena in Kasungu National Park, Malawi

Covariate	Source	Sampling range (mean)	Hypothesized effect	Supporting evidence
Hyena detection	Camera trap	1 (detection) 0 (nondetection)	−^a^	Swanepoel et al. 2015; Balme, Miller, et al. 2017
Leopard detection	Camera trap	1 (detection) 0 (nondetection)	+^b^	Balme, Miller, et al. 2017
Distance to water (km)	GIS	0.03–10.45 (3.35)	+	Watts & Holekamp, 2009; Havmøller et al. 2019
Distance to park border (km)	GIS	0.78–14.38 (7.99)	−	Woodroffe & Ginsberg, 1998; Balme et al. 2010
Preferred prey detection	Camera trap	1 (detection) 0 (nondetection)	+	Höner et al. 2005; Balme et al. 2019
Habitat type*	Observation	1 (open) 0 (closed)	−^a^ +^b^	Balme et al. 2007; Watts & Holekamp, 2009

Note

The hypothesized effect on large carnivore detection is indicated, alongside supporting evidence for the predicted effect.

^a^ Effect on leopard detection.

^b^ Effect on hyena detection.

* Hypothesized effect is based on habitat openness.

Vegetation cover, hunting strategy, and landscape features can all impact carnivore detection rates, as predators select areas optimal for increased prey density, heightened vulnerability to predation, and their preferred hunting method (i.e., denser cover for ambush, open habitat for endurance; Balme et al., 2007; Watts & Holekamp, 2009). We used a binary variable for habitat type (Strampelli et al., 2018), where each camera site was designated as either “open,” where at least one side of the trail was bordered by open grassland, or “closed,” where both sides of the trail were bordered by miombo woodland.

For distance‐based covariates (i.e., distance to water, distance to park border), the Euclidian distance (km) between each camera trap and the chosen feature were extracted in QGIS v.2.18.16 (QGIS Development Team, 2020). As KNP has no buffer zone and no continual fencing, distance to park border was selected as a suitable covariate to test for human disturbance. Clearance for agricultural land and the lack of a buffer zone means human settlements often begin at the KNP park boundary (Munthali & Mkanda, 2002). We reasoned that distance to park border was, therefore, a suitable covariate to incorporate both the impact of edge effects (Woodroffe & Ginsberg, 1998) and the proximity to human settlements (Balme et al., 2010).

Generalized linear mixed‐effect models were conducted in R v.3.6.3 (R Development Core Team, 2020), using package “lme4” (Bolker et al., 2009). We removed one camera trap that malfunctioned shortly after being set from the analyses. There was no significant collinearity (*r* < 0.5 for all pairwise comparisons) between continuous covariates, and therefore, none were excluded from model selection. We aggregated data from all survey years and included year as a random effect to compensate for temporal variability. Camera station ID was also fitted as a random effect to control for repeated measures between sites (Cusack et al., 2017). All possible combinations of detection covariates were modeled for both leopard and hyena, with only selected prey species differing between model sets (see Supplementary Material A found on Dryad Data Repository for full candidate lists). We used an information theoretic approach whereby models were ranked on their Akaike information criterion (AICc, corrected for small sample sizes) and models with ΔAICc < 2 considered to have strong support and selected for model averaging (Burnham & Anderson, 2002). From the final set of candidate models (ΔAICc < 2), average *β*‐coefficient estimates were obtained using the “MuMIn” package (Barton, 2020). Individual covariates were deemed significant when 85% confidence limits did not pass through zero, following Arnold (2010). The importance of individual covariates for predicting large carnivore detection was assessed using the summed model weights (Σw) of all models in the final candidate set. There was no evidence of overdispersion (*ĉ* > 1.1) across models, which was calculated as the ratio of the sum of the squared Pearson residuals to the residual degrees of freedom (Harrison, 2014).

### 2.4 Time‐to‐event analysis

We used time‐to‐event analyses to examine leopard and hyena response to sympatric carnivores and preferred prey species across survey seasons (Balme et al., 2019; Cusack et al., 2017). Prey species were kept as defined for co‐detection modeling. For each reference detection (defined as a photographic capture of a chosen species, e.g., leopard), we calculated the minimum time to capture the species of proximal interest (e.g., hyena) at the same camera station. Any occasion where a reference detection was followed by another detection of the reference species was removed from the analyses. The calculated times between reference and proximal detections were then aggregated into 24‐hr sampling intervals, with interval limits of five days before or after the reference detection (*n* = 10 days). For each 24‐hr interval (*n* = 10 intervals), we then calculated an observed detection probability by dividing the number of proximal detections in each interval period by the total number of detections in the survey year for the species of proximal interest.

Expected distributions of proximal detection were randomly simulated by sampling activity patterns and capture rates of the proximal species, to generate new dates and times, which were then compared to the original, unchanged, reference detections (Cusack et al., 2017). From 1,000 random iterations of proximal detection, we obtained expected values of detection probability for each 24‐hr interval, which were then compared to the observed probability using standard two‐tailed permutation tests, using the package “ade4” (Dray & Siberchicot, 2020). Analyses could not be conducted for the 2016 survey, or between leopard sexes, as sample sizes were too small.

### 2.5 Temporal activity

Camera trap images from all survey years were used to estimate daily activity levels (percentage of time spent active over the 24‐hr daily cycle) and degree of temporal overlap between large carnivore species and, for leopard, between individual sexes. Data for both large carnivore species were combined across survey years for the final analyses. We tested data for each species (and individual sexes for leopard) for differences between survey years to ensure no bias between individual years (Supplementary Material B found on Dryad Data Repository). To determine whether activity patterns were significantly different to a random distribution over the circadian cycle, we performed a Hermans–Rasson test (Landler et al., 2019) on temporal data for both leopard and hyena, using the package “CircMLE” (Fitak & Johnsen, 2017). We used the time and date stamp from all photographic captures to determine animal activity. All models were fitted to clock time as surveys were conducted during the same survey period (between May and October each year), and daylight variance is limited at latitudes below 20° (Vazquez et al., 2019). To reduce bias and overrepresentation of activity at certain times of the day, only one photographic capture was used for analysis when time stamps were within 30 min of each other, unless unique pelage patterns confirmed different individuals were photographed. We performed analyses when species presented a minimum of thirty images accumulated in each survey year, as small sample sizes can bias activity estimations and misrepresent activity levels (Rowcliffe et al., 2014). We conducted analyses using the “overlap” (Meredith & Ridout, 2016) and “activity” (Rowcliffe, 2019) packages in R v3.6.3 (R Development Core Team, 2020).

Overall activity (i.e., the distribution of animal activity throughout the day) was estimated using the Kernel circular density function in “activity” (Rowcliffe et al., 2014; Santos et al., 2019). Overlap of activity was quantified using the coefficient of overlap (Δ), which varies from 0 (no overlap) to 1 (complete overlap) (Santos et al., 2019). The Δ_4_ estimator was used for all species included in the analyses as all sample sizes were ≥75, and Δ_4_ is considered the most robust estimator for this sample size (Meredith & Ridout, 2014; Ridout & Linkie, 2009). To estimate confidence intervals for activity levels, we simulated 10,000 smoothed bootstrap samples. Pairwise comparisons of bootstrapped activity patterns were then tested for significant differences in the “activity” package, using a Wald statistic on a chi‐square distribution with one degree of freedom (Rovero & Zimmermann, 2016).

## 3 RESULTS

### 3.1 Camera trap results

We completed 5,990 camera trap nights across 92 camera trap stations in KNP between 2016 and 2018, with 702 photographic captures of large carnivore species and 854 prey species (Table 2). Sufficient sample sizes for temporal analyses were recorded for leopard and hyena (>30 captures in each survey year). The presence of one subadult male lion was recorded in 2017, while one wild dog was recorded in 2017 and again in 2018 (determined by a unique pelage pattern), confirming the absence of resident lion and wild dog populations in KNP.

**TABLE 2:** List of species detected and yearly and total counts from camera trap surveys between 2016 and 2018 in Kasungu National Park, Malawi

Order	Scientific name	Common name	2016 captures	2017 captures	2018 captures	Total captures
Carnivora	*Panthera pardus*	Leopard	48	116	115	279
	*Crocuta crocuta*	Spotted hyena	113	148	133	394
	*Panthera leo*	Lion	0	11	0	11
	*Lycaon pictus*	African wild dog	0	9	9	18
Artiodactyla	*Sylvicapra grimmia*	Common duiker	22	42	63	127
	*Tragelaphus sylvaticus*	Bushbuck	4	7	7	18
	*Tragelaphus strepsiceros*	Greater kudu	1	6	17	24
	*Phacochoerus africanus*	Warthog	4	9	12	25
	*Potamochoerus larvatus*	Bushpig	13	48	36	97
Lagomorpha	*Lepus victoriae*	Savanna hare	25	110	45	180
Rodentia	*Hystrix africaeaustralis*	Cape porcupine	24	166	158	348
Primates	*Papio cynocephalus*	Yellow baboon	5	23	7	35

Capture totals are provided for all large carnivores recorded and the prey species of leopard and spotted hyena that were chosen for spatiotemporal analyses.

### 3.2 Co‐detection analyses

#### 3.2.1 Leopard

Four models (ΔAICc < 2) were selected from the final set of 11 candidate models (combined AICc weights >0.95) for model averaging (Table 3). There was no evidence of overdispersion (*ĉ* = 0.90) in the most parameterized model. Detection of prey (*β* = 0.443 ± 0.162, 85% CI = 0.210–0.676), proximity to water (*β* = 0.311 ± 0.110, 85% CI = 0.152–0.470), and detection of hyena (*β* = 0.310 ± 0.178, 85% CI = 0.053–0.567) were positive predictors of leopard detection. Prey detection and proximity to water were the best predictors of leopard detection (Σw = 1.0 for both).

**TABLE 3:** Model selection for binomial generalized linear mixed models predicting the likelihood of leopard detection at camera stations in Kasungu National Park, Malawi, across all survey years (2016, 2017, and 2018) during a given 5‐day sampling occasion

Model	*K* ^a^	AICc	ΔAICc	*W*_i_	Cum. *W*_i_	Log likelihood
Hyena + prey + water	6	1,120.34	0.00	0.31	0.31	−554.13
Prey + water	5	1,121.38	1.05	0.18	0.49	−555.67
Hyena + prey + water + habitat	7	1,121.91	1.58	0.14	0.63	−553.91
Hyena + prey + water + border	7	1,122.34	2.00	0.11	0.74	−554.12
Prey + water + habitat	6	1,123.01	2.68	0.08	0.82	−555.47
Prey + water + border	6	1,123.37	3.03	0.07	0.89	−555.65
Hyena + prey + water + border + habitat	8	1,123.90	3.57	0.05	0.94	−553.89
Prey + water + border + habitat	7	1,124.98	4.64	0.03	0.97	−555.44
Hyena + water	5	1,125.96	5.63	0.02	0.99	−557.96
Hyena + prey	5	1,126.85	6.51	0.01	1.00	−558.40

Parameter	*β*‐coefficient	*SE*±	Lower 85%	Upper 85%	Σw (%)
Prey*	0.443	0.162	0.210	0.676	1.0
Water*	0.311	0.110	0.152	0.470	1.0
Hyena*	0.310	0.178	0.053	0.567	0.75
Border	0.017	0.108	−0.138	0.172	0.19
Habitat	−0.144	0.223	−0.465	0.177	0.15

Models were ranked according to Akaike weights (*W_i_*) based on the Akaike information criterion for small samples (AICc), and cumulative model weight is also presented (Cum. *W_i_*). Models with AICc differences (ΔAICc) < 2 were averaged, and *β*‐coefficient estimates, with associated standard error (*SE*±), 85% confidence limits, and summed model weights (Σw), were presented.

^a^ Number of parameters in the model.

* Indicates parameter had a significant effect on leopard detection as 85% confidence limits exclude zero.

### 3.3 Hyena

Five models (ΔAICc < 2) were identified for model averaging from the final set of 22 candidate models (AICc weights >0.95; Table 4). There was no evidence of overdispersion (*ĉ* = 0.93) in the most parameterized model. The detection of prey (*β* = 0.366 ± 0.163, 85% CI = 0.131–0.601) and leopard (*β* = 0.303 ± 0.182, 85% CI = 0.041–0.566) was positive predictors of hyena detection, and both terms had high model support (preferred prey, Σw = 1.00; leopard, Σw = 0.78).

**TABLE 4:** Model selection for binomial generalized linear mixed models predicting the likelihood of hyena detection at camera stations in Kasungu National Park, Malawi, across all survey years (2016, 2017, and 2018) during a given 5‐day sampling occasion

Model	*K* ^a^	AICc	ΔAICc	*W*_i_	Cum. *W*_i_	Log likelihood
Prey + leopard	5	1,245.60	0.00	0.19	0.19	−617.78
Prey	4	1,246.48	0.87	0.12	0.31	−619.22
Prey + leopard + habitat	6	1,247.08	1.47	0.09	0.40	−617.50
Prey + leopard + border	6	1,247.35	1.75	0.08	0.48	−617.64
Prey + leopard + water	6	1,247.50	1.89	0.07	0.55	−617.71
Prey + habitat	5	1,247.97	2.37	0.06	0.61	−618.96
Prey + border	5	1,248.18	2.58	0.05	0.66	−619.07
Prey + water	5	1,248.44	2.84	0.05	0.71	−619.20
Leopard	4	1,248.75	3.14	0.04	0.75	−620.36
Prey + leopard + habitat + water	7	1,248.88	3.27	0.04	0.79	−617.39

Parameter	*β*‐coefficient	*SE*±	Lower 85%	Upper 85%	Σw (%)
Prey*	0.366	0.163	0.131	0.601	1.00
Leopard*	0.303	0.182	0.041	0.566	0.78
Habitat	0.178	0.247	−0.178	0.533	0.16
Border	0.060	0.121	−0.114	0.235	0.14
Water	−0.041	0.120	−0.213	0.131	0.13

Models were ranked according to Akaike weights (*W*_i_) based on the Akaike information criterion for small samples (ΔAICc), and cumulative model weight is also presented (Cum. *W*_i_). Models with AICc differences (ΔAICc) < 2 were averaged, and *β*‐coefficient estimates, with associated standard error (SE ±) and 85% confidence limits, were presented. Only the ten highest ranking models are presented here.

^a^ Number of parameters in the model.

* Indicates parameter had a significant effect on hyena detection as 85% confidence limits exclude zero.

### 3.4 Time‐to‐event analysis

#### 3.4.1 Leopard–hyena

**FIGURE 2:**
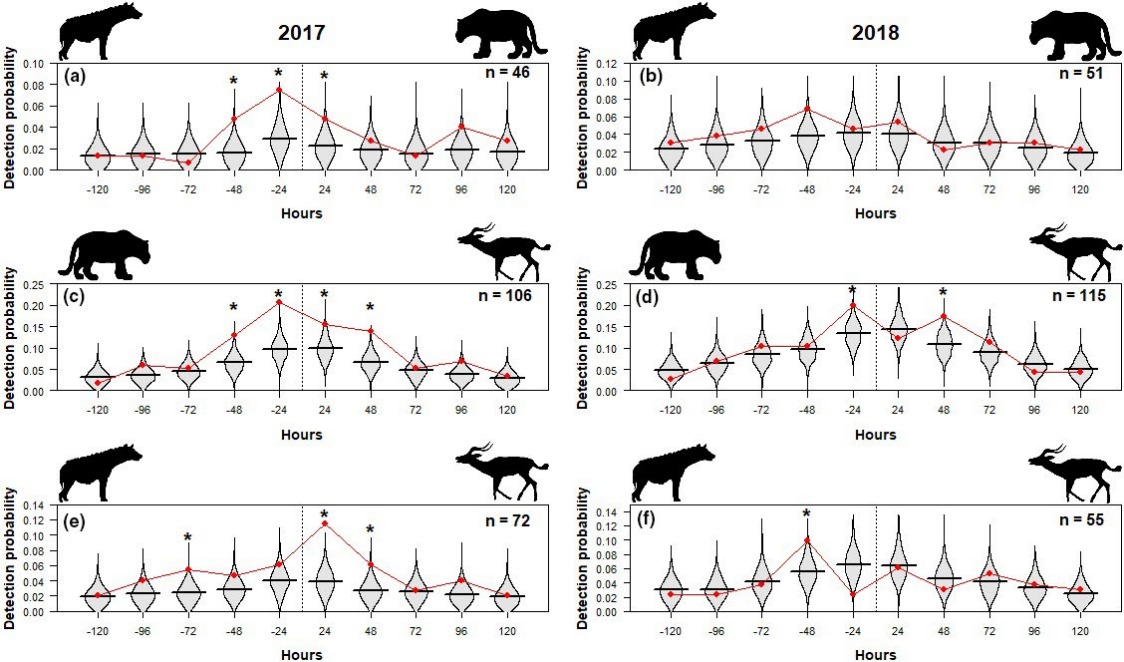
The observed (red) and expected (gray) probability of detecting hyena after a leopard capture in 2017 (a) and 2018 (b), leopard after a prey species capture in 2017 (c) and 2018 (d), and hyena after a prey species capture in 2017 (e) and 2018 (f), at the same sampling site within five days before and after in Kasungu National Park, Malawi. Asterisks (*) above expected distributions, obtained from 1,000 random simulations of capture events for the corresponding species, indicate days for which observed detection rates were significantly different (*p* < 0.05) to expected values. Sample sizes, from which observed detection probabilities were calculated, are given for each year

Compared to expected detection probability distributions, hyena were more likely to be detected in the 24 hr after a leopard event during the 2017 survey (*p* < 0.05; Figure 2). In 2017, leopard capture events were significantly more likely when hyena had been captured in the previous 24 (*p* < 0.01) and 48 (*p* < 0.05) hours. In the 2018 survey, there was no significant bias in detection shown by either species.

#### 3.4.2 Leopard–prey

Leopard detections were higher 24 (*p* < 0.05; Figure 2) and 48 (*p* < 0.001) hours after, and 24 and 48 hr (both *p* < 0.001) before a prey detection in 2017. Leopard detections were significantly higher 24 hr (*p* < 0.05) before and 48 hr (*p* < 0.05) after a prey detection in 2018.

#### 3.4.3 Hyena–prey

Hyena response to a prey detection was comparable to leopard response in the 2017 survey, with increased detections 24 (*p* < 0.001; Figure 2) and 48 (*p* < 0.05) hours after prey species detections. Hyena detections were higher within 72 hr (*p* < 0.05) before a prey detection in 2017. Hyena detections were higher than expected within 48 hr (*p* < 0.05) before prey species detection in 2018.

#### 3.4.4 Temporal activity

**FIGURE 3:**
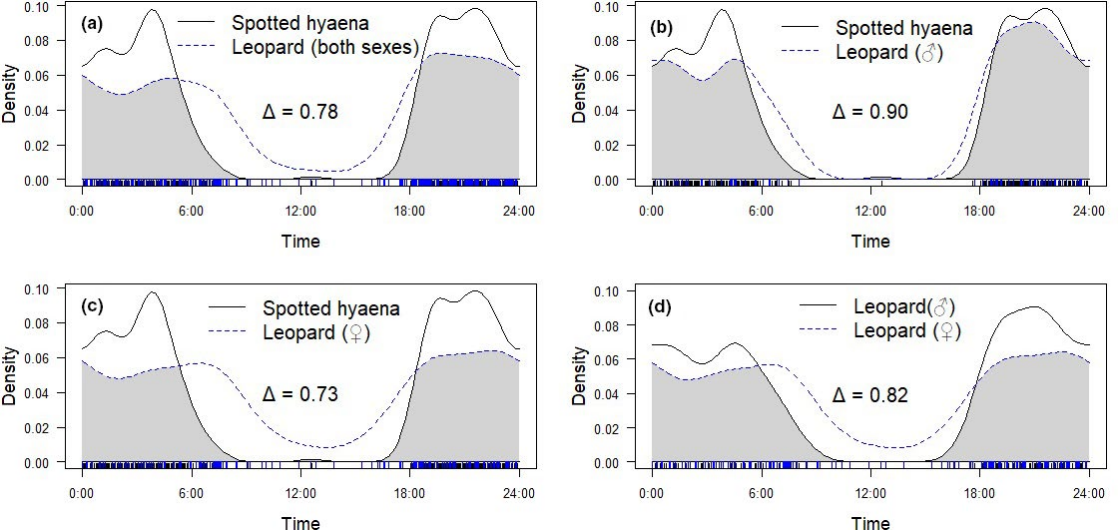
Temporal overlap in activity patterns between (a) spotted hyena and leopard (both sexes); (b) spotted hyena and male leopard; (c) spotted hyena and female leopard; and (d) male and female leopard. Temporal activity patterns are compiled from surveys conducted in Kasungu National Park, Malawi, between 2016 and 2018. Coefficient of overlap (Δ) for each pairwise comparison is displayed, and shaded areas represent temporal overlap

Overall activity (estimated proportion of time spent active over the daily cycle) was 0.57 (*SE* = 0.05) for leopard (both sexes), 0.46 (*SE* = 0.06) for male leopard, and 0.65 (*SE* = 0.06) for female leopard and 0.42 (*SE* = 0.03) for hyena (Table 5). The Hermans–Rasson test confirmed that both leopard and hyena had activity patterns that were significantly different from random (*p* < 0.001 for all). We observed an overlap average of Δ = 0.78 for leopard–hyena, Δ = 0.9 for male leopard–hyena, Δ = 0.73 for female leopard–hyena, and Δ = 0.82 for male leopard–female leopard (Figure 3). The lowest coefficient of overlap observed was between female leopard and hyena. Leopard showed higher levels of diurnal activity with peaks at dawn and dusk, while hyena showed higher levels of strictly nocturnal activity, with peaks before dawn and after dusk.

**TABLE 5:** Estimates of proportion of time active for large carnivore species in Kasungu National Park, Malawi, estimated from the distribution of camera trapping photographs over the daily cycle

Species	*N*	Estimate	SE	95% CI
Leopard (both sexes)*	273	0.573	0.048	0.473–0.659
Leopard (♂)	77	0.459	0.056	0.312–0.525
Leopard (♀)	170	0.649	0.056	0.504–0.723
Spotted hyena	385	0.423	0.027	0.359–0.465

*N* is the number of photographic captures, and estimate is the overall activity with standard error (SE) and 95% confidence intervals (95% CI).

* Includes images of leopards that could not be sexed but identified to species level.

There was a 15% difference in overall temporal activity levels between leopard (both sexes) and hyena in KNP (Wald *χ^2^* = 7.39, *df* = 1, *p* < 0.01, Table 6). However, when individual leopard sexes were compared with hyena, there was only a 4% difference in overall activity levels between male leopard and hyena (Wald *χ^2^* = 0.34, *df* = 1, *p* = 0.55). Female leopards were active for 23% more of the daily cycle than hyena (Wald *χ^2^* = 13.05, *df* = 1, *p* < 0.001) and nearly 20% more active than male leopards (Wald *χ^2^* = 5.76, *df* = 1, *p* < 0.05).

**TABLE 6:** Estimates of difference in activity between large carnivore species in Kasungu National Park, Malawi, from the distribution of camera trapping photographs over the diel activity schedule

Species interaction	Difference	*SE*	*W*	*p*
Leopard (both sexes)–spotted hyena	0.151	0.055	7.39	0.007
Leopard (♂)–leopard (♀)	0.190	0.079	5.763	0.016
Leopard (♂)–spotted hyena	0.037	0.062	0.346	0.556
Leopard (♀)–spotted hyena	0.227	0.063	13.048	<0.001

Bootstrapped activity patterns, with 10,000 smoothed bootstrap samples, were compared using Wald statistic (*W*) on a chi‐square distribution with one degree of freedom in order to test for significance (*p*) at the 5% level.

## 4 DISCUSSION

Spatiotemporal dynamics play an important role in facilitating coexistence between the large carnivore guild, yet little is known about these dynamics in human‐altered landscapes (Sévêque et al., 2020). In protected areas where anthropogenic disturbance disrupts community structure, competition between remaining carnivores is predicted to increase (Périquet et al., 2015). We explored spatiotemporal partitioning between leopard and hyena in a modified guild where they are the only competing large carnivores, providing a novel habitat in which to test theories on guild dynamics. Our results indicate that prey availability and the presence of competing carnivores positively influence the spatiotemporal dynamics of both leopard and hyena. In the absence of a resident lion population and the depleted prey base in KNP, these shared drivers of spatiotemporal behavior increase the likelihood of costly interactions and could have negative consequences for large carnivore demographics.

Our findings show that prey detection is a significant predictor of detection for both hyena and leopard, supporting our predictions and in accordance with previous studies (Höner et al., 2005; Périquet et al., 2015; Ramesh et al., 2017; Searle et al., 2020). Leopard detection was also explained by proximity to water, as observed in previous studies (Balme et al., 2007; Havmøller et al., 2019). This finding supports our hypothesis that leopard space use is primarily driven by prey presence in KNP, as prey species are commonly associated with riparian areas, and these areas provide adequate cover for the leopards’ preferred ambush technique (Balme et al., 2007). Confirming our prediction, co‐detection and time‐to‐event analyses showed a mutually positive influence between hyena and leopard, as recorded by Balme et al. (2019). Given their competitive dominance and propensity for kleptoparasitism (Balme, Miller, et al., 2017), the influence of leopard presence on hyena space use likely indicates the additional benefits of high spatiotemporal overlap for hyena. In similar areas of Africa, where prey abundance is depleted, there is evidence that dietary overlap increases between large carnivores (Creel et al., 2018). As prey presence was a significant predictor of leopard and hyena detection, it may be that both species are responding to the same environmental cue (i.e., prey availability) resulting in increased co‐detection rates.

The high spatial overlap of leopard and hyena in KNP, combined with mutual drivers of detection, is likely to increase interaction between the two species. Despite the inherent risk of interaction with dominant competitors (i.e., lion and hyena), previous studies have shown that intraguild competitors often have little bearing on leopard spatiotemporal dynamics (Balme, Pitman, et al., 2017; Miller et al., 2018; Rafiq, Jordan, Wilson, et al., 2020; Strampelli et al., 2018). In the absence of spatiotemporal responses, leopards are often reliant on behavioral adaptability, such as tree caching and dietary plasticity, to support intraguild coexistence (Voigt et al., 2018; Balme et al., 2019). In KNP, this is evident for male leopards, as we recorded high temporal overlap between male leopard and hyena. This finding challenges our prediction that both leopard sexes would display temporal avoidance of hyena, as observed by Havmøller, Jacobsen, Scharff, et al. (2020). In contrast, female leopards displayed different temporal activity patterns to hyena. Kleptoparasitism from hyena has been shown to negatively impact reproductive success of female leopard and female leopards suffer higher rates of kleptoparasitism, compared with males (Balme, Miller, et al., 2017). As such, increased interaction with hyenas presents a greater risk for female leopards and could explain the temporal partitioning. Furthermore, male leopards are more likely to display tree‐caching behavior than female conspecifics (Balme, Miller, et al., 2017; Stein et al., 2015), which could facilitate greater coexistence with hyena. Tree caching would be less effective for female leopard due to the threat of intraspecific kleptoparasitism (Balme, Miller, et al., 2017), and this could lead female leopards to adopt the additional mechanism of temporal partitioning found in this study (Miller et al., 2018).

Our results support Havmøller, Jacobsen, Scharff, et al. (2020), who recorded temporal differences between leopard sexes and increased levels of female diurnal activity compared with males. These findings highlight the importance of incorporating sex into pairwise behavioral comparisons. Increased interaction with male conspecifics heightens the risk of kleptoparasitism and infanticide for female leopards, and observed temporal differences could be a mechanism to minimize these costly encounters (Balme et al., 2013, 2017; Swanepoel et al., 2015). Miller et al. (2018) hypothesized that temporal segregation between leopard and interspecific competitors could increase at sites of reduced prey abundance, due to higher rates of resource sharing, which may explain the sex‐specific and interspecific differences in temporal activity observed here. In addition, female leopards can exhibit wider dietary niches than male conspecifics, often displaying more opportunistic feeding strategies and predating on smaller‐bodied prey items (e.g., Voigt et al., 2018). The wider dietary plasticity of female leopards could be an additional mechanism to facilitate coexistence, and further investigation of leopard sex‐specific dietary specialization in KNP would improve our knowledge of intraguild dynamics and niche partitioning strategies.

Female leopard daily activity levels were 19%–23% higher than those of male leopard and hyena. These extended periods of diel activity may increase the likelihood of interaction with intraguild competitors and anthropogenic threats (e.g., road traffic, human activity), thus heightening exposure to potential sources of mortality (Havmøller, Jacobsen, Scharff, et al., 2020; Rizzuto et al., 2018). The greater energetic costs imposed by higher activity levels may reduce reproductive success and overall fitness (Rizzuto et al., 2018; Wilmers et al., 2017), creating cascading demographic effects. Further research is required to assess the potential impacts of intraguild competition and depleted prey on female leopard fitness and reproductive success.

There was no effect of proximity to park boundary or habitat type on detection of leopard or hyena. These findings highlight the ability of both species to persist throughout the protected area, which is encouraging for local conservation management. We acknowledge that the coarse scale on which habitat was assessed here may not be sufficient to identify fine‐scale habitat preferences. Previous studies have highlighted the higher tolerance of hyena (Mkonyi et al., 2018) and leopard (Petracca et al., 2019; Strampelli et al., 2018) to human presence, compared with other large carnivores (Everatt et al., 2019). Our results provide further evidence of the species’ adaptability in areas of close proximity to human settlement. However, our temporal analyses suggest that hyena activity is largely restricted to nocturnal movements, which is considered an early response to high levels of human disturbance (Holekamp & Dloniak, 2010; Kolowski et al., 2007).

We acknowledge that our results are restricted to KNP and further efforts to quantify spatiotemporal behaviors in modified carnivore guilds would be beneficial to inform carnivore conservation management in human‐altered landscapes. Malawi offers an interesting avenue for such studies, as several protected areas have seen similar reductions in large carnivore and prey populations (Mésochina et al., 2010; van Velden et al., 2020). In this study, camera trap placement was focused on roads and trails to optimize capture rates for large carnivores. Despite this, we are confident our findings are representative of carnivore habitat use in KNP, as road systems play an integral role in carnivore space use (Rafiq, Jordan, Meloro, et al., 2020). In addition, since large carnivore densities are low in KNP (Davis et al., 2021), it is also the only viable, noninvasive method for gathering large amounts of data to quantify carnivore behavior (Rowcliffe et al., 2014). However, the use of road networks could have reduced prey species capture rates, as these areas increase exposure to predation risk and human activity, potentially underrepresenting aspects of observed predator–prey interaction (Havmøller, Jacobsen, Scharff, et al., 2020; Oriol‐Cotterill et al., 2015).

Camera trap density and length of sampling occasion for co‐detection and time‐to‐event models could have reduced precision of estimates. While overall detections were similar for leopard and hyena in 2017 and 2018, interactive behaviors may be underrepresented in 2018 as only half the number of camera trap sites were deployed, due to logistical reasons. Although aggregating detection events into larger bins may impact the accuracy of parameter estimates (as models are sensitive to changes in temporal scale; see Cusack et al., 2017), this practice is commonly used for large carnivores that have naturally low detection rates (e.g., Abade et al., 2018; Strampelli et al., 2018). Future studies could look to increase the density of camera traps deployed to yield higher capture rates, and this may allow for shorter temporal scales to be used. However, given the low densities of large carnivores in KNP it is unlikely that an occasion length shorter than 24 hr could be applied. The deployment of GPS collars with high sampling rates, as in Rafiq, Jordan, Wilson, et al. (2020), could be of greater benefit to gather fine‐scale data on carnivore activity and encounter rates.

Improved law enforcement efforts and ongoing reintroductions of prey species could increase prey abundance in KNP (IFAW, 2020). Under these conditions, and with the absence of a competing lion population, hyena numbers could quickly rise, as observed by M’soka et al. (2016) in Liuwa Plains, Zambia. Conversely, leopard population recovery is gradual and reproductive success is naturally low (Balme et al., 2013; Balme, Robinson, et al., 2017). Increased hyena clan size would have direct benefits for food acquisition and hyena cub survival (Höner et al., 2005), potentially exacerbating current levels of interspecific competition. In response to increased competition, leopards are likely to adapt their spatiotemporal behavior and may switch to smaller prey items (Comley et al., 2020; du Preez et al., 2017) or be forced into suboptimal habitat (e.g., low prey abundance, edge habitats; Vanak et al., 2013). Additional behavioral adaptations could have negative consequences for population recovery. For example, Comley et al. (2020) hypothesized that the decreasing leopard population in Selati Game Reserve, South Africa, was attributable to high levels of interspecific competition with the resident, much larger, hyena population. As such, close monitoring of large carnivore densities and intraguild dynamics is required in KNP to assess the impact of ongoing conservation initiatives.

We have shown that leopard and hyena coexist in KNP, with male leopard and hyena showing significant spatiotemporal overlap, while female leopards exhibit temporal partitioning to mitigate potential interactions with intra‐ and interspecific competitors. Whether the behavioral responses of female leopards are sufficient to maintain reproductive success and long‐term population viability is unknown. Our results show that prey occurrence is a significant predictor of leopard and hyena detection. Therefore, protecting remaining prey populations should be a management priority to conserve the resident carnivore guild. Further understanding of the drivers of spatiotemporal behaviors can help alleviate the challenges caused by changing niches and shifts in carnivore community dynamics (Rafiq, Jordan, Wilson, et al., 2020). As protected areas are subject to increasing levels of anthropogenic disturbance (Jones et al., 2018), further research of large carnivore spatiotemporal dynamics will be imperative to maintain carnivore coexistence and to implement effective long‐term conservation strategies.

## CONFLICT OF INTEREST

The authors declare that they have no conflict of interest.

## AUTHOR CONTRIBUTIONS

**Robert Davis:** Conceptualization (equal); Data curation (lead); Formal analysis (lead); Funding acquisition (supporting); Investigation (lead); Resources (supporting); Writing‐original draft (lead); Writing‐review & editing (equal). **Richard Yarnell:** Conceptualization (equal); Funding acquisition (supporting); Resources (supporting); Supervision (lead); Writing‐review & editing (equal). **Louise K. Gentle:** Conceptualization (equal); Funding acquisition (supporting); Resources (supporting); Supervision (equal); Writing‐review & editing (equal). **Antonio Uzal:** Conceptualization (equal); Funding acquisition (supporting); Resources (supporting); Supervision (equal); Writing‐review & editing (equal). **William O. Mgoola:** Writing‐review & editing (equal). **Emma Stone:** Conceptualization (equal); Funding acquisition (lead); Project administration (lead); Resources (lead); Supervision (equal); Writing‐review & editing (equal).

## ETHICAL APPROVAL

The ethical approval was granted by the Nottingham Trent University and Department of National Parks and Wildlife Malawi.

## Data Availability

The data that support the findings of this study are available in the Dryad Data Repository at https://doi.org/10.5061/dryad.n2z34tmwm.
